# Radiomics-Based Differential Diagnosis of Radicular Cysts and Apical Granulomas on CBCT Images Using RadC-CNN Architecture

**DOI:** 10.3390/diagnostics16101428

**Published:** 2026-05-07

**Authors:** Bilgün Çetin, Derya İçöz, Kevser Dinç, İsmail Kayadibi

**Affiliations:** 1 Department of Oral and Maxillofacial Radiology, Faculty of Dentistry, Selcuk University, 42250 Konya, Turkey; deryayilmaz@selcuk.edu.tr (D.İ.); kews-@hotmail.com (K.D.); 2 Department of Management Information Systems, Faculty of Economic and Administrative Sciences, Afyon Kocatepe University, 03200 Afyonkarahisar, Turkey; ikayadibi@aku.edu.tr

**Keywords:** radiomic features, CBCT images, radicular cyst, periapical granuloma, deep learning

## Abstract

**Background/Objectives:** This study aims to evaluate the diagnostic performance of radiomic features derived from cone-beam computed tomography (CBCT) images in differentiating radicular cysts (RC) from periapical granulomas (PG). The study also compares the performance of traditional machine learning (ML) algorithms with a novel deep learning (DL) model, Radiomics Cyst Convolutional Neural Network (RadC-CNN). **Methods**: CBCT images of 98 patients (55 RC, 43 PG), confirmed by histopathological diagnosis, were retrospectively analyzed. Lesions were semi-automatically segmented in 3D Slicer, and 48 radiomic features were extracted. Features with high inter-observer agreement (Intraclass Correlation Coefficient ICC ≥ 0.80) were included in the analysis. Statistical tests and classification models (Decision Tree, K-Nearest Neighbors, Support Vector Machine) were used, and performance was compared to that of the proposed RadC-CNN architecture. **Results:** Among the 34 features with sufficient reliability, 18 showed statistically significant differences between RC and PG (*p* < 0.05). Shape, first-order, and texture-based features, including the Gray Level Co-occurrence Matrix (GLCM), Gray Level Run Length Matrix (GLRLM), Gray Level Size Zone Matrix (GLSZM), and Neighboring Gray Tone Difference Matrix (NGTDM), were extracted. The RadC-CNN model demonstrated superior classification performance with an accuracy of 90%, sensitivity of 90%, and precision of 91.3%, outperforming all traditional ML algorithms. **Conclusions**: CBCT-based radiomic analysis, particularly when combined with DL techniques like RadC-CNN, offers a promising non-invasive approach to distinguish RC from PG.

## 1. Introduction

Periapical or periradicular lesions generally arise as a host immune response to microbial infection within the root canal system, aiming to prevent the spread of infection to surrounding tissues. These lesions are characterized by localized inflammation that may lead to periapical tissue destruction and bone resorption [1]. Among the most common periapical lesions are radicular cysts (RCs), periapical granulomas (PGs), and apical periodontitis, which typically appear as apical radiolucencies on intraoral periapical radiographs [2]. Although these lesions share similar radiographic characteristics due to infection-induced bone resorption, their biological behavior and treatment approaches differ [3,4].

Histopathological examination remains the gold standard for differentiating cystic from non-cystic periapical lesions; however, this approach requires surgical intervention [1]. Clinically, PGs often respond well to non-surgical endodontic treatment following the removal of the inflammatory stimulus within the root canal system, whereas RCs may persist or continue to grow independently of the initial inflammatory trigger and frequently require surgical management, such as cystectomy [5,6,7]. Therefore, the ability to distinguish between these lesions using non-invasive imaging methods is of considerable clinical importance in order to guide treatment decisions and avoid unnecessary surgical procedures [1,3,4,5,7,8].

Advances in imaging technologies, particularly cone-beam computed tomography (CBCT), have significantly improved the visualization of periapical structures in oral and maxillofacial radiology. In recent years, the increasing availability of high-resolution imaging data has facilitated the application of radiomics and artificial intelligence (AI) techniques for medical image analysis [9,10].

Radiomics enables the extraction of large numbers of quantitative features from medical images, describing characteristics such as intensity, shape, and texture of regions of interest, thereby allowing images to be analyzed beyond subjective visual interpretation [10,11,12,13]. These features may reveal underlying imaging biomarkers that are not readily detectable by conventional radiographic assessment [14].

The integration of AI methods, including machine learning (ML) and deep learning (DL), has further expanded the potential of radiomics in medical imaging. Traditional ML algorithms, such as decision trees (DT), support vector machines (SVM), and k-nearest neighbors (KNN), have been widely used for classification tasks in radiomics-based studies. However, these approaches generally rely on manually extracted features and may have limitations in capturing complex and high-dimensional imaging patterns. In contrast, deep learning models, particularly convolutional neural networks (CNNs), can automatically learn hierarchical feature representations and have demonstrated strong performance in image-based classification tasks. Therefore, combining radiomics with DL approaches may enhance the ability to detect subtle imaging patterns and improve diagnostic accuracy [15,16,17,18,19,20].

The aim of this study was to evaluate the performance of the Radiomics Cyst Convolutional Neural Network (RadC-CNN) model for differentiating radicular cysts from periapical granulomas through a comparative analysis with commonly used ML algorithms, including KNN, SVM, and DT. Such an approach may contribute to the non-invasive differentiation of these lesions, potentially supporting clinical decision-making and improving treatment planning before invasive procedures are considered.

## 2. Materials and Methods

This study was designed and conducted at the Department of Oral and Maxillofacial Radiology, Faculty of Dentistry, Selçuk University. The Non-Interventional Clinical Research Ethics Committee of Selçuk University Faculty of Dentistry approved this study with decision number 2025/12.

### 2.1. Sample Size Calculation

According to the reference study [21], the sample size for this study was calculated using G-Power (version 3.1.9.7, Heinrich-Heine-Universität Düsseldorf, Düsseldorf, Germany) for the *t*-test. With an effect size of 0.68 (d = 0.68), 80% power (1 − β = 0.80), and 5% margin of error (α = 0.05), the minimum required sample size for each lesion group was determined to be 36. In this study, archival screening yielded 55 data points for RC and 43 for PG that met the inclusion criteria.

### 2.2. Patients

Between October 2018 and December 2025, patients diagnosed with odontogenic cysts and possessing CBCT data in the archives of the Department of Oral and Maxillofacial Radiology were retrospectively reviewed. Patients were included if their histopathological diagnoses were definitively confirmed as either RC or PG based on the presence or absence of epithelium, and if their preoperative CBCT images clearly depicted the entire boundaries of the lesion. However, images with severe metal artifacts that compromised image quality, as well as lesions smaller than 10 mm in size that were inadequate for segmentation, were excluded from the study. A total of 98 patients were included, comprising 55 patients with a confirmed diagnosis of RC and 43 patients with a confirmed diagnosis of PG, based on consistent clinical, radiographic, and histopathological findings.

### 2.3. CBCT Image Acquisition and Segmentation

All CBCT scans were acquired using a single CBCT system and standard protocol (Instrumentarium Dental, Palo DEx Group Oy, Nahkelantie 160 FI-04300 Tuusula, Finland) with an exposure setting of 89 kVp and a current range of 4–12 mA.

**Figure 1:**
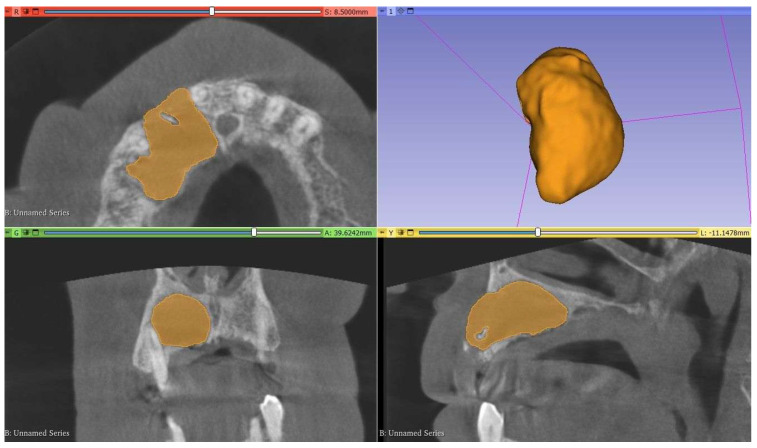
Example segmentation of a radicular cyst using the 3D Slicer program.

CBCT images were imported into 3D Slicer version 5.6.2, an open-source software available at https://www.slicer.org, in Digital Imaging and Communications in Medicine (DICOM) format. The images were standardized to a voxel volume of 0.25 × 0.25 × 0.25 mm^3^ using the ‘Resample Scalar Volume’ module with cubic B-spline interpolation. Semi-automatic full segmentation of all RCs and PGs was performed by an oral radiologist with six years of experience, utilizing a predefined threshold range based on the mean gray scale intensity of the lesions. The threshold range was specifically adjusted for each lesion to achieve optimal differentiation from the surrounding bone tissue. Complete lesion segmentation was achieved by manually refining each slice layer by layer in every plane (Figure 1). Another oral radiologist with eight years of experience re-segmented 20 randomly selected lesions (approximately 20% of the data) using the same method. After the segmentation was completed, the Laplacian of Gaussian filter (σ = 0.5) was applied from the ‘Smoothing’ section to reduce noise and enhance edges and details.

### 2.4. Radiomic Features Extraction

**Figure 2:**
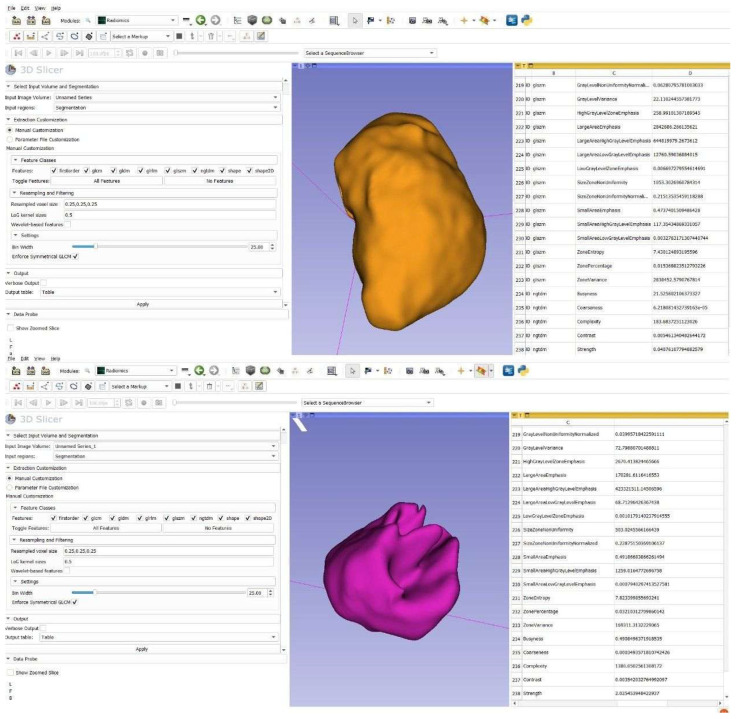
Extracted features from the radiomic module of the example segmentation. The upper lesion is a radicular cyst (orange), and the lower lesion is an apical granuloma (purple).

After completing the segmentation and filtering processes, Radiomic features were derived through the ‘Radiomics’ module integrated into the 3D Slicer software (version 5.8.3 Brigham and Women’s Hospital, Boston, MA, USA) (Figure 2). The original versions of shape-based features were recorded, while the first-order and textural features were saved with the LoG-filtered version. A total of 48 features were recorded and analyzed. Five shape-based features (Voxel volume, Sphericity, Elongation, Flatness, Max 3D diameter), five first-order features (Energy, Entropy, Kurtosis, Skewness, Variance), nine GLCM (Gray Level Co-occurrence Matrix) features (Auto-correlation, Cluster prominence, Cluster shade, Contrast, Correlation, Difference variance, IMC1, IMC2, Sum average), twelve GLRLM (Gray Level Run Length Matrix) features (Gray level non-uniformity, Gray level variance, High gray level run emphasis, Long run emphasis, Long run high gray level emphasis, Long run low gray level emphasis, Low gray level run emphasis, Run length non-uniformity, Run percentage, Short run emphasis, Short run high gray level emphasis, Short run low gray level emphasis), twelve GLSZM (Gray Level Size Zone Matrix) features (Gray level non-uniformity, Gray level variance, High gray level zone emphasis, Large area emphasis, Large area high gray level emphasis, Large area low gray level emphasis, Low gray level zone emphasis, Size zone non-uniformity, Small area emphasis, Small area high gray level emphasis, Small area low gray level emphasis, Zone percentage), and five NGTDM (Neighborhood Gray Tone Difference Matrix) features (Busyness, Coarseness, Complexity, Contrast, Strength) features were analyzed.

### 2.5. Statistical Analysis

Statistical analyses were performed using IBM SPSS (Statistical Package for the Social Sciences) Statistics Version 22 (Armonk, NY, USA). To assess the repeatability of the extracted radiomic features, inter-observer agreement was evaluated in the re-segmented lesions using the Intraclass Correlation Coefficient (ICC). Features with an ICC of 0.80 or higher were selected for further statistical analysis.

Descriptive statistics, including mean, minimum, maximum, and standard deviation, as well as frequency analysis, were applied for the lesion groups. The normality of the data distribution was assessed using the Shapiro–Wilk test. Quantitative data with a normal distribution were analyzed using the Independent Samples *t*-test, while non-normally distributed data were analyzed using the Mann–Whitney U test. A *p*-value of <0.05 was considered statistically significant.

### 2.6. Model Training and Evaluation

**Figure 3:**
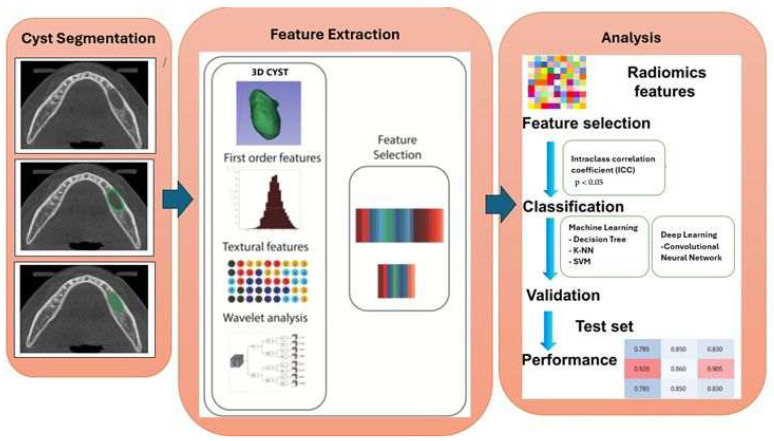
Flow diagram of the proposed methodology.

Following the completion of segmentations and the extraction of radiomic features, the AI model proceeded to the training and testing phases. To reduce dimensionality and minimize the risk of overfitting, a two-step feature selection approach was applied. First, the reproducibility of radiomic features was assessed using the intraclass correlation coefficient (ICC), and only features with ICC ≥ 0.80 were retained. Subsequently, statistical analysis was performed to identify features that showed significant differences between RC and PG, and only these discriminative features were used for model training. The flowchart outlining the entire proposed methodology is presented in Figure 3.

In this study, two different validation strategies were employed to evaluate the model’s performance in a more comprehensive and reliable manner. Firstly, 90% of the dataset was allocated for training and 10% for testing. This fixed split method was used to assess the model’s overall accuracy on a limited test set. The details of the radiomics dataset obtained by radiomics analysis on CBCT images are given in Table 1.

**Figure 4:**
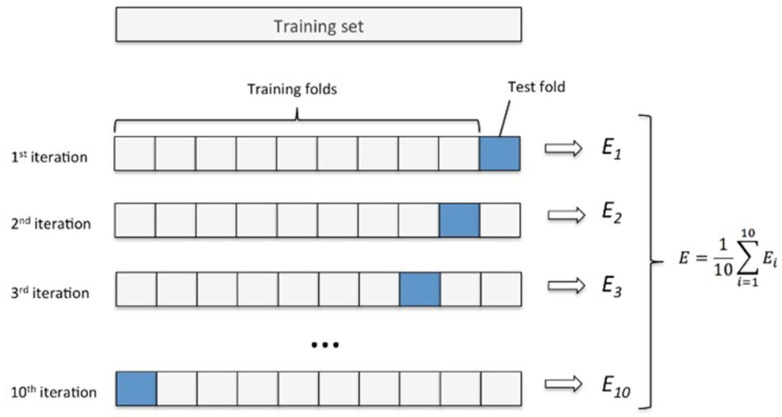
Schematic representation of the 10-fold cross-validation process used to evaluate model performance.

In addition, 10-fold cross-validation was applied to establish a more robust basis for assessing the model’s generalizability and stability. In this approach, the dataset was divided into 10 equal subsets; each subset was used once as the test set while the remaining nine were used for training (Figure 4). In this way, all samples were utilized both as training and testing data, enabling a detailed observation of the model’s performance across different data.

In this study, ML algorithms such as DT, KNN, SVM, and XGBoost, along with the DL-based RadC-CNN architecture, were trained on the training subset of the dataset using radiomic feature sets. The trained models were subsequently evaluated on the test subset, enabling a comprehensive performance assessment of both ML and DL techniques. To ensure experimental reproducibility, the random seed was fixed across all training procedures of both DL and ML techniques.

### 2.7. Proposed RadC-CNN Architecture

**Figure 5:**
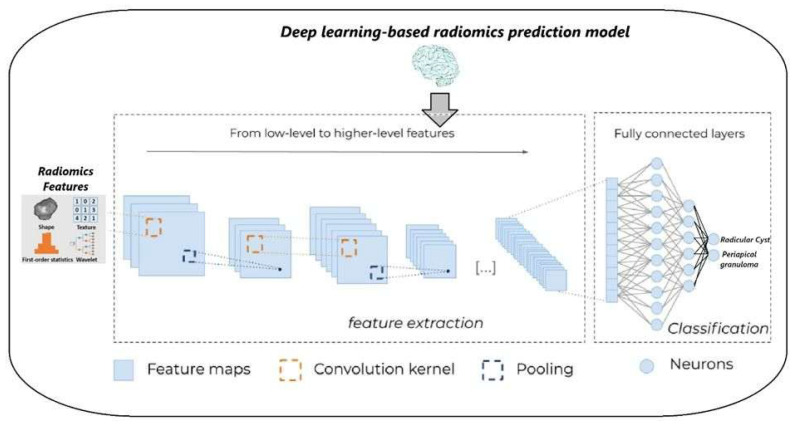
DL-based RadC-CNN architecture.

In this study, a DL-based radiomics prediction model, RadC-CNN, is proposed for the detection of lesions such as RC and PG using radiomic features. The architecture of the proposed model is detailed in Figure 5.

The proposed RadC-CNN architecture is a tailored deep CNN specifically designed to perform multi-class classification on structured one-dimensional feature vectors, such as radiomic features extracted from CBCT scans. To ensure compatibility with the convolutional architecture, input vectors of dimensionality [numFeatures × 1 × 1] are reshaped into four-dimensional tensors with dimensions [numFeatures, 1, 1, N], where N denotes the number of observations. This formatting enables the use of two-dimensional convolutional layers while functionally implementing a one-dimensional convolutional process. The network begins with a convolutional layer comprising 512 filters of size 3 × 1, which capture localized patterns across the feature dimension. This is immediately followed by a batch normalization layer that stabilizes learning by normalizing activations, and a rectified linear unit (ReLU) activation layer that introduces non-linearity by zeroing out negative values. This initial block enables the model to learn low-level feature abstractions. The subsequent convolutional block includes 256 filters of the same size, enabling the extraction of increasingly abstract representations. This block also incorporates batch normalization and ReLU activation, followed by a max-pooling layer with a window size of 2 × 1 and a stride of 1. The pooling operation reduces the spatial dimension along the feature axis, highlighting the most salient activations while mitigating overfitting. A third convolutional block mirrors the structure of the second, with 256 filters of size 3 × 1, and includes normalization, activation, and pooling layers, allowing for deeper hierarchical feature extraction. The fourth and final convolutional block utilizes 64 filters to further refine the high-level semantic representations while significantly reducing the computational load. Each convolutional block successively transforms the feature space into increasingly abstract and informative representations, while the combination of normalization and pooling improves generalization and training efficiency. Following the convolutional layers, the extracted feature maps are flattened and passed through a sequence of fully connected layers. The first dense layer consists of 1024 neurons and is followed by a dropout layer with a rate of 0.1 to prevent overfitting. This is followed by a second dense layer with 256 neurons and a subsequent dropout layer with a rate of 0.05. The final classification layer is a fully connected layer with a number of neurons equal to the number of target classes. A softmax layer at the output converts the raw class scores into probability distributions, enabling multi-class classification. This architecture is optimized for radiomics applications characterized by high-dimensional feature vectors and relatively limited sample sizes. By combining deep convolutional feature extraction with regularization techniques such as dropout and batch normalization, RadC-CNN is capable of learning robust, generalizable feature representations suitable for radiomic feature classification tasks in medical imaging analysis.

Initially, the dataset underwent preprocessing to address missing and erroneous values, followed by z-score normalization to standardize feature distributions prior to being input into the RadC-CNN. Subsequently, the RadC-CNN architecture was trained using predefined hyperparameters and tested on a designated subset of the dataset. The model’s performance was assessed using a confusion matrix, and overall accuracy was documented.

Additionally, ML algorithms such as K-NN, SVM, XGBoost, and DT were trained and tested on the radiomic feature dataset to facilitate a comparative performance analysis. The performance metrics for these algorithms were computed and reported, and a comparison table was provided to illustrate the results of the proposed method in contrast to conventional ML algorithms.

### 2.8. Hyperparameter Tuning

The tuning of hyperparameters is a critical factor in optimizing the performance of CNNs [17], as it directly affects training efficiency, generalization capability, and overfitting control. In this study, key hyperparameters of the proposed RadC-CNN architecture were carefully determined through empirical experimentation based on validation performance.

The proposed architecture was trained using the Adam optimization algorithm with a learning rate of 1 × 10^−3^, a gradient decay factor of 0.9, and a squared gradient decay factor of 0.999 [22]. A mini-batch size of 4 and a maximum of 100 epochs were employed, considering the limited size of the radiomics dataset. To enhance generalization and prevent overfitting, L2 regularization (1 × 10^−4^) and early stopping with a patience value of 15 epochs were applied. The training process was accelerated using a GPU environment. Model performance was monitored using the accuracy metric, and validation was conducted using a hold-out validation set.

### 2.9. Performance Metrics

**Figure 6:**
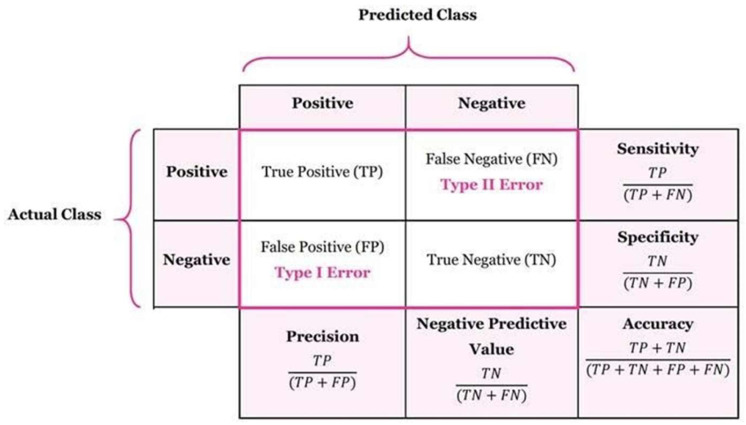
A confusion matrix is represented by mathematical equations of performance metrics.

In this research, the effectiveness of the ML algorithms and RadC-CNN architecture used in the proposed methodology was evaluated using the confusion matrix, which is an important tool in classification tasks [23]. The confusion matrix enables the calculation of several important performance metrics such as accuracy, sensitivity, specificity, precision, and F1 score (Figure 6). It provides a comprehensive insight into the agreement of the model’s predictions with the actual class labels and consists of four main components:

True Positives (TP): Instances in which the model correctly identifies a positive case, meaning the model’s prediction of a positive outcome is accurate.

True Negatives (TN): Instances where the model correctly identifies a negative case, indicating that the model’s prediction of a negative outcome is correct.

False Positives (FP): Instances where the model incorrectly predicts a positive outcome, meaning the model’s prediction is positive, but the actual outcome is negative.

False Negatives (FN): Instances where the model incorrectly predicts a negative outcome, indicating that the model’s prediction is negative, but the actual outcome is positive.

## 3. Results

The age and gender information of the individuals whose images were used in this study are presented in Table 2. A total of 98 images were segmented and analyzed, including 55 RC and 43 PG. Among the radiomic features, 48 different parameters were recorded. Features with an ICC of 0.80 or higher were used for statistical analysis and ML model development. Among these, 34 radiomic features showed good to excellent agreement between the two observers.

RCs were most frequently observed in the maxillary anterior region with 24 cases, followed by the mandibular posterior region with 21 cases. Five lesions were located in the mandibular anterior region and five in the maxillary posterior region. PGs were most commonly found in the maxillary anterior region with 19 cases, followed by the mandibular posterior region with 11 cases. Eight lesions were located in the mandibular anterior region and five in the maxillary posterior region. Lesion size characteristics, including mean lesion volume (voxel volume) and maximum three-dimensional diameter (max. 3D diameter), are presented in Table 3.

### 3.1. Shape-Based Features

Among the non-textural features, shape-based characteristics were analyzed, including voxel volume (mm^3^), elongation, flatness, max. 3D diameter (mm), and sphericity. Of these parameters, voxel volume and max. 3D diameter showed a statistically significant difference between RC and PG (*p* < 0.001 and *p* = 0.005, respectively). The values for RC were found to be significantly higher than those for PG (Table 3).

### 3.2. First-Order Features

First-order features, energy, entropy, kurtosis, and variance were included in further analyses due to the high agreement between the two observers. Among these features, a significant difference was found between the two lesion groups, except for kurtosis. Energy exhibited higher values in the RC group, whereas entropy and variance were higher in the PG group compared to RC (Table 3).

### 3.3. Second or Higher Order Features

Among the GLCM features, cluster prominence, cluster shade, contrast, difference variance, and IMC2 were included in further analyses due to the high agreement between the two observers. The analysis revealed that among these GLCM features, only contrast showed a significant difference between RC and PG lesions. According to this finding, contrast values were higher in the PG group compared to the RC group.

The GLRLM features gray level non-uniformity, gray level variance, long run emphasis, run length non-uniformity, run percentage, and short run emphasis were selected for further statistical analyses due to their high agreement. The results indicated that all GLRLM features, except for gray level variance, exhibited a significant difference between the groups (*p* < 0.05). Gray level non-uniformity, long run emphasis, and run length non-uniformity values were higher in the RC group, whereas run percentage and short run emphasis values were greater in the PG group.

The GLSZM features, including gray level non-uniformity, gray level variance, large area emphasis, large area high gray level emphasis, large area low gray level emphasis, low gray level zone emphasis, size zone non-uniformity, small area emphasis, small area low gray level emphasis, and zone percentage, were selected due to their high agreement. According to the results, statistically significant differences were found in the features of gray level non-uniformity, large area emphasis, large area high gray level emphasis, large area low grey level emphasis, and size zone non-uniformity. For all these features, the mean values in the RC group were higher than those in the PG group.

Finally, the NGTDM features busyness, coarseness, complexity, and contrast were similarly selected. Statistically significant differences were observed in busyness and coarseness. The mean values of both features were higher in the RC group compared to the PG group.

### 3.4. Performance Evaluation with ML Algorithms and RadC-CNN Architecture

**Figure 7:**
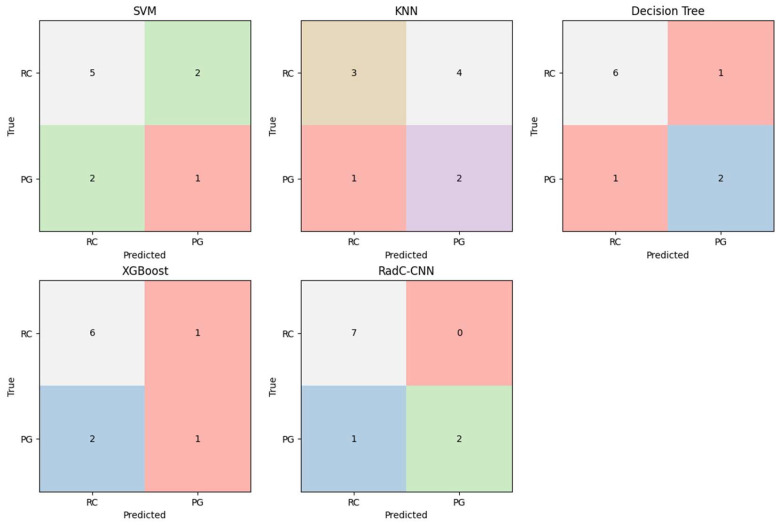
Confusion matrices of obtained with ML and DL techniques.

In this study, the proposed RadC-CNN architecture was trained on the radiomics dataset and evaluated on the test set. Additionally, conventional machine learning models, including KNN, SVM, Decision Tree (DT), and XGBoost, were trained and tested under the same conditions. The performance comparison based on confusion matrix metrics is presented in Table 4, while the corresponding confusion matrices are shown in Figure 7.

RadC-CNN achieved the highest performance among all evaluated models, with an accuracy of 90%, sensitivity of 90%, precision of 91.3%, and an F1-score of 89.3%. The DT model ranked second, with an accuracy of 80%, specificity of 87.5% for the RC class, and both precision and recall values of 79.7%. XGBoost demonstrated moderate performance, achieving an accuracy of 70%, sensitivity of 70%, precision of 67.5%, and an F1-score of 68.0%, although its specificity remained relatively low. SVM achieved an accuracy of 60% and exhibited relatively lower performance compared to DT and XGBoost. KNN showed the weakest performance, with an accuracy of 50%, a high false-positive rate of 57.1% for the RC class, and a low specificity of 42.9%.

To analyze the overall performance of the RadC-CNN architecture on the radiomics dataset, a 10-fold cross-validation was additionally performed, and the resulting findings are presented in Table 5.

The results of the 10-fold cross-validation indicate that the model demonstrated a basic level of overall success in distinguishing between the two lesions. An average accuracy of 60% is noteworthy for a model trained with a limited number of features and samples. This suggests that the model is capable of capturing general patterns even with a relatively simple architecture.

## 4. Discussion

In this study, radiomics-based ML models and a DL approach were compared for the differentiation of RC and PG. The primary aim was to evaluate the diagnostic performance of traditional ML algorithms (Decision Tree, SVM, and KNN) and a CNN-based model (RadC-CNN) using the same radiomic feature set. The results indicated that the DL model generally achieved higher performance across several evaluation metrics compared with the ML algorithms. This finding may suggest that DL-based models have a greater potential to capture complex patterns within radiomic data.

Histopathological examination, an invasive method, remains the gold standard for distinguishing between RC and PG [21,24]. However, establishing a definitive diagnosis before surgery is important, as it may influence the treatment approach. Therefore, several studies have investigated the differentiation of these lesions using various imaging and analytical methods [1,3,5,7,8,21,24,25,26]. The imaging modalities used include direct radiographs [7,8,27], ultrasonography (USG) [1,25], magnetic resonance imaging (MRI) [3,6], CT [21], and CBCT [5,26].

To the best of our knowledge, the use of CBCT-based radiomic features for the differentiation of RC and PG has not been previously reported. The study most closely related to this research is that of Yomtako et al. [21], who evaluated radiomic features derived from multislice CT images for the differential diagnosis of RC and PG. In contrast, the present study aimed to investigate whether radiomic features extracted from CBCT images—an imaging modality more commonly used in dental practice—could support the differentiation of these lesions by applying both ML and DL approaches.

Another relevant study is the one conducted by De Rosa et al. [5], which evaluated texture parameters derived from CBCT images for the differential diagnosis of RC and PG. Regions of interest (ROIs) were defined within the lesions, and 11 texture parameters were extracted. These parameters were assessed using receiver operating characteristic (ROC) analysis to determine their discriminative ability. The results showed that five texture parameters had significant predictive value in differentiating between RC and PG. The authors concluded that texture parameters obtained from CBCT images may provide useful information for the differential diagnosis of periapical lesions. Consistent with these findings, in the present study, 18 of the 34 radiomic features included in the analysis showed statistically significant differences between the RC and PG groups. This observation suggests that radiomic analysis may capture quantitative differences in intralesional characteristics. Such differences may reflect variations in tissue composition and structural heterogeneity within the lesions, although the direct biological interpretation of individual radiomic features remains limited. Therefore, radiomics-based approaches may provide supportive quantitative information for lesion characterization; however, further validation with larger datasets is required before clinical implementation can be considered.

In our study, 18 radiomic features showed statistically significant differences between RC and PG lesions, suggesting subtle imaging characteristics that may not be readily discernible to the naked eye. Among these, voxel volume and maximum 3D diameter were significantly higher in the RC group compared with the PG group. This observation may be consistent with the expected biological behavior of RCs, which, due to their cystic nature and fluid content, often present as larger and more expansive lesions. In contrast, PGs may appear as smaller lesions with less well-defined borders, resulting from chronic inflammatory infiltration [4,27]. However, it should be noted that the direct biological interpretation of individual radiomic features remains limited, and these associations should therefore be interpreted with caution.

In the first-order features, energy was significantly higher in RCs, while entropy and variance were higher in PGs. Energy refers to the sum of the squares of the pixel or voxel intensities in an image. A high value indicates that the intensities are more homogeneous, meaning there is less variation, and may suggest an active lesion. The higher Energy values in RCs may reflect the more homogeneous intensity distribution observed in cystic structures, which are typically characterized by fluid content and relatively uniform internal composition [28]. On the other hand, entropy is a feature that measures the irregularity or randomness of intensity values in an image, while variance quantifies the extent to which pixel intensities deviate from the mean intensity [10]. Therefore, the elevated entropy and variance values observed in PGs may indicate a higher degree of signal heterogeneity, which could stem from varying degrees of inflammation, fibrosis, and necrotic tissue components. PG lesions are typically more heterogeneous in nature, containing inflammation, fibrosis, and fluid components, and thus may exhibit higher entropy and variance values. This may reflect the more irregular structure of PGs. These findings may be consistent with the known histopathological differences between the two lesions and suggest the potential of first-order statistics to distinguish inflammatory and cystic periapical pathologies.

Among the GLCM features, only contrast showed a significant difference between the groups, with higher values observed in PGs. Contrast measures local intensity variation [29] and typically increases in heterogeneous tissues [30]. The elevated contrast in PGs may reflect their more heterogeneous internal structure, which includes inflammatory cells, connective tissue, and variable vascularization.

GLRLM is used to quantify the length of consecutive pixels with the same gray level in a given image, providing information about the distribution of homogeneous runs and regional texture patterns within the lesion [30]. Metrics such as short-run emphasis, long-run emphasis, gray-level non-uniformity, and run-length non-uniformity characterize the distribution and variability of gray-level runs and are widely used to describe texture patterns in medical images [31]. In the present study, most GLRLM features exhibited significant differences between lesion types, suggesting that run-length–based texture characteristics are sensitive to the internal structural organization of periapical lesions. Higher gray-level non-uniformity, long-run emphasis, and run length non-uniformity observed in RCs may reflect the presence of larger and more homogeneous fluid-filled cavities typical of cystic lesions, leading to longer and more uniform gray-level runs. In contrast, the higher run percentage and short run emphasis detected in PGs indicate more frequent gray-level transitions and finer texture patterns, which may correspond to the histologically heterogeneous composition of granulomatous inflammatory tissue.

Several GLSZM features (particularly gray level non-uniformity, large area emphasis, and size zone non-uniformity) were significantly higher in RCs, indicating that these lesions consist of larger, more homogeneous regions with similar gray levels. This finding is consistent with the cystic nature of RCs, which are typically presented as having a central radiolucent area surrounded by a thin cortical boundary [32]. In contrast, PGs, which generally lack a well-organized internal structure, exhibited lower values for these parameters, reflecting their tissue complexity and heterogeneity.

When examining the NGTDM features that reflect the differences between a voxel and its neighbors [33], the significant increase in busyness and coarseness values in RCs may indicate sharper intensity transitions and a coarser texture pattern. While this may initially appear counterintuitive, it could be related to the presence of well-defined cystic boundaries and higher signal contrast between the lesion content and the surrounding tissues. On the other hand, PGs may exhibit more gradual intensity transitions due to widespread inflammatory infiltration, leading to lower values in these features. Overall, these results suggest that NGTDM-based features may provide complementary information for characterizing the internal texture heterogeneity of periapical lesions.

This study evaluated four different classification approaches for distinguishing between RC and PG: SVM, DT, KNN, and the RadC-CNN architecture. The resulting models were compared based on their weighted average performance metrics and confusion matrices. Among the evaluated methods, the RadC-CNN model achieved the highest performance across most metrics, generally outperforming the other approaches. The model reached an accuracy of 90%, with a sensitivity of 90% and a precision of 91.3%, demonstrating a relatively balanced classification of both RC and PG classes. Additionally, the F1-score of 89.3% reflects the model’s ability to maintain overall class balance. These results suggest that the RadC-CNN may be capable of learning higher-level representations from radiomic features through its deep layers, while dropout and batch normalization mechanisms may help control overfitting.

Recent reviews have highlighted the increasing application of deep learning and radiomics in CT and CBCT imaging for the diagnosis and classification of maxillofacial diseases. Deep learning models, particularly CNN-based architectures, have demonstrated high diagnostic performance and, in some cases, have achieved results comparable to or exceeding those of human experts. These observations are consistent with the findings of the present study, where the RadC-CNN model demonstrated comparatively higher performance than traditional machine learning algorithms [9]. Nevertheless, further validation using larger and independent datasets remains necessary before clinical implementation can be considered.

Previous studies have also explored the use of CBCT-based radiomics combined with machine learning for the classification of jaw lesions. For example, Sha et al. [34] developed several machine learning models using CBCT radiomic features to differentiate odontogenic cysts, odontogenic keratocysts, and ameloblastomas. Among the evaluated algorithms, an ensemble VotingClassifier achieved the best performance with an overall accuracy of approximately 71% in the test set. In the present study, the Decision Tree model achieved a higher accuracy of 80% for distinguishing radicular cysts from periapical granulomas. However, direct comparison between the studies should be interpreted cautiously because the previous study involved a larger dataset and a multi-class classification problem, whereas the present study focused on a binary classification task with a smaller sample size.

Overall, the confusion matrices and metric analyses reveal that the RadC-CNN model shows a more consistent pattern of classification performance compared with the other models. Traditional methods such as SVM, KNN, and DT appear more sensitive to dataset size and feature dimensionality, which may affect their stability in small datasets. Rather than indicating definitive superiority, these findings suggest that DL-based approaches such as RadC-CNN may offer promising performance for radiomics-based classification tasks. These developments are consistent with the broader transformation toward Healthcare 5.0, where artificial intelligence and data-driven technologies are increasingly integrated into healthcare systems to enhance diagnostic accuracy and support clinical decision-making [35].

From a clinical perspective, although the DL model may support the differentiation between PA and RC and help reduce unnecessary surgical procedures, lesions classified as granulomas should still be carefully monitored through periodic clinical and radiographic follow-up. Such follow-up is important to evaluate the response to endodontic treatment and to identify any potential progression or cystic transformation over time.

This study has several limitations. Firstly, its single-center and retrospective design may reduce sample diversity and limit the generalizability of the findings. Additionally, the dataset used in the study includes a limited number of cases, which may not reflect the actual frequency of these two lesions in clinical practice. The restricted sample size is primarily due to the strict inclusion criteria, such as requiring both lesions to be confirmed by pathological reports and explicitly indicating the presence of epithelium. While these criteria ensured diagnostic accuracy, they inevitably reduced the number of eligible cases. Consequently, the limited dataset may have affected the statistical power of the analyses and may also increase the risk of overfitting in machine learning models.

Moreover, the semi-automatic segmentation of lesions is subject to observer variability, which could compromise the reliability of the extracted radiomic features. Although efforts were made to maintain consistency during the segmentation process, observer-dependent variability may still influence the reproducibility of radiomic features. Therefore, future studies are recommended to employ fully automatic and standardized segmentation methods to minimize observer-dependent variability and improve the reproducibility of radiomic feature extraction. Furthermore, the developed models were not externally validated using an independent dataset, which may limit the assessment of their generalizability. Future studies, including larger, multicenter datasets and independent validation cohorts, would help improve the robustness and clinical applicability of radiomics-based models. In addition, integrating clinical follow-up data may enhance the classification accuracy of radiomics-based models and contribute to improved model validity.

## 5. Conclusions

This study explored the potential of radiomic analysis applied to CBCT images as a non-invasive approach for the differential diagnosis of RC and PG. Among the evaluated classification models, the RadC-CNN architecture achieved the highest diagnostic performance, suggesting its ability to capture subtle textural and structural differences between lesions. Based on our findings, shape-based features such as voxel volume and maximum 3D diameter, first-order features including energy, entropy, and variance, as well as several second-order features derived from GLCM, GLRLM, GLSZM, and NGTDM matrices, showed potential discriminatory value in distinguishing RC from PG.

The integration of these radiomic features with the RadC-CNN model demonstrated promising classification performance, highlighting the potential benefit of combining radiomics with deep learning approaches. However, these findings should be interpreted with caution, and further validation with larger and independent datasets is required before clinical implementation can be considered. Nevertheless, radiomics-based models may offer supportive information for diagnostic decision-making, particularly in cases where conventional imaging findings are inconclusive.

## Figures and Tables

**Table 1:** Details about the Radiomics dataset (RC: Radicular cyst, PG: Periapical Granuloma).

Radiomics Dataset			
Lesion	Train	Test	Total
**RC**	48	7	55
**PG**	40	3	43
**Total**	88	10	98

**Table 2:** Distribution of the lesions by gender and age.

	Gender						Age		
	Female		Male		Total		Min.	Max.	Mean ± SD
	n	%	n	%	n	%			
**RC**	18	18.4	37	37.8	55	56.1	12	94	35.71 ± 14.89
**PG**	23	23.4	20	20.4	43	43.9	12	66	33.4 ± 14.23
**Total**	41	41.8	57	58.2	86	100	12	94	34.69 ± 14.57

RC: radicular cyst; PG: periapical granuloma; Min: minimum; Max: maximum; SD: standard deviation.

**Table 3:** Radiomics features of the RC and PG.

	RC Mean ± SD	PG Mean ± SD	*p* Value
	**Shape-based features**		
Voxel volume (mm^3^)	2915.51 ± 3378.51	1210.53 ± 968.06	˂0.001 *
Elongation	0.75 ± 0.15	0.74 ± 0.13	0.747
Flatness	0.55 ± 0.17	0.55 ± 0.15	0.897
Max. 3D diameter (mm)	24.26 ± 9.40	18.90 ± 6.71	0.001 *
Sphericity	0.73 ± 0.7	0.72 ± 0.8	0.586
	**First-order features**		
Energy	1,183,027,618.12 ± 1,185,914,734.07	887,899,021.26 ± 781,818,171.08	0.018 *
Entropy	3.46 ± 0.32	3.62 ± 0.36	0.026 *
Kurtosis	4.84 ± 1.68	4.73 ± 2.31	0.273
Variance	6237 ± 2420.93	7830.16 **±** 4675.48	0.047 *
	**GLCM**		
Cluster prominence	6960.11 ± 5305.16	15,199.12 ± 5178.49	0.325
Cluster shade	221.94 ± 167.17	300.37 ± 397.68	0.630
Contrast	2.30 ± 0.80	2.90 ± 1.79	0.037 *
Difference variance	1.29 ± 0.54	1.56 ± 1.01	0.396
lmc2	0.94 ± 0.10	0.94 ± 0.15	0.660
	**GLRLM**		
Gray level non-uniformity	11,843.31 ± 15,342.50	4554.70 ± 3634.12	0.003 *
Gray level variance	12.00 ± 4.55	13.97 ± 8.38	0.437
Long run emphasis	4.63 ± 1.45	3.88 ± 1.25	0.010 *
Run length non-uniformity	51,002.27 ± 51,789.06	25,758.65 ± 17,987.96	0.001 *
Run percentage	0.62 ± 0.06	0.66 ± 0.07	0.004 *
Short-run emphasis	0.73 ± 0.05	0.76 ± 0.06	0.017 *
	**GLSZM**		
Grey level non-uniformity	224.71 ± 271.84	126.72 ± 117.64	0.020 *
Grey level variance	23.72 ± 7.76	30.22 ± 21.18	0.140
Large area emphasis	1,577,748.36 ± 2,956,264.52	454,021.55 ± 551,337.17	0.023 *
Large area high gray level emphasis	490,238,899.89 ± 1,266,290,713.98	114,723,543.85 ± 204,139,812.73	0.012 *
Large area low gray level emphasis	7842.33 ± 16,775.58	5222.62 ± 9595.42	0.045 *
Low gray level zone emphasis	0.009 ± 0.007	0.012 ± 0.014	0.780
Size zone non-uniformity	1006.68 ± 1494.98	602.68 ± 702.60	0.007 *
Small area emphasis	0.50 ± 0.06	0.49 ± 0.08	0.818
Small area low gray level emphasis	0.005 ± 0.003	0.005 ± 0.004	0.583
Zone percentage	0.030 ± 0.027	0.033 ± 0.024	0.583
	**NGTDM**		
Busyness	11.60 ± 13.84	6.75 ± 8.62	0.003 *
Coarseness	1.67 ± 3.07	0.14 ± 0.95	0.001 *
Complexity	267.89 ± 153.19	373.48 ± 383.66	0.499
Contrast	0.011 ± 0.006	0.13 ± 0.006	0.151

* Statistically significant (*p* < 0.05). GLCM: gray-level co-occurrence matrix, GLRLM: gray-level run length matrix, GLSZM: gray-level size zone matrix, NGTDM: neighboring gray-tone difference matrix.

**Table 4:** Performance comparison with performance metrics of the RadC-CNN architecture and ML methods.

Method	Accuracy (%)	Sensitivity (%)	Specificity (%)	Precision (%)	F1 Score (%)
DT	80.0	79.7	87.5	79.7	79.7
SVM	60.0	61.3	71.4	61.3	61.3
KNN	50.0	50.0	42.9	60.8	51.1
XGBoost	70	70	49	67.5	68.0
**RadC-CNN**	**90.0**	**90.0**	**80.0**	**91.3**	**89.3**

**Table 5:** Performance results with cross-validation of the RadC-CNN architecture.

Fold	Accuracy	Sensitivity	Specificity	Precision	F1 Score
**1**	0.5556	0.2500	0.8000	0.5000	0.3333
**2**	0.5000	0.2500	0.6667	0.3333	0.2857
**3**	0.7000	0.2500	1.0000	1.0000	0.4000
**4**	0.6000	0.0000	1.0000	0.0000	0.0000
**5**	0.7000	0.7500	0.6667	0.6000	0.6667
**6**	0.6000	0.5000	0.6667	0.5000	0.5000
**7**	0.5000	1.0000	0.0000	0.5000	0.6667
**8**	0.7000	1.0000	0.4000	0.6250	0.7692
**9**	0.6000	0.4000	0.8000	0.6667	0.5000
**10**	0.5556	0.7500	0.4000	0.5000	0.6000
**Mean**	0.6011	0.5150	0.6400	0.5225	0.4722

## Data Availability

The data analyzed during the current study are not publicly available due to privacy and ethical restrictions but are available from the corresponding author on reasonable request.

## Abbreviations

The following abbreviations are used in this manuscript:

**CBCT**	Cone-Beam Computed Tomography
**CT**	Computed Tomography
**GLCM**	Gray Level Co-occurrence Matrix
**GLRLM**	Gray Level Run Length Matrix
**GLSZM**	Gray Level Size Zone Matrix
**NGTDM**	Neighborhood Gray Tone Difference Matrix
**AI**	Artificial Intelligence
**RadC-CNN**	Radiomics Cyst Convolutional Neural Network
**ML**	Machine Learning
**DL**	Deep Learning
